# A Peptidoglycan Fragment Triggers β-lactam Resistance in *Bacillus licheniformis*

**DOI:** 10.1371/journal.ppat.1002571

**Published:** 2012-03-15

**Authors:** Ana Amoroso, Julien Boudet, Stéphanie Berzigotti, Valérie Duval, Nathalie Teller, Dominique Mengin-Lecreulx, André Luxen, Jean-Pierre Simorre, Bernard Joris

**Affiliations:** 1 Centre d'Ingénierie des Protéines, Institut de Chimie B6A, Sart-Tilman, Université de Liège, Liège, Belgium; 2 Cátedra de Microbiología, Facultad de Farmacia y Bioquímica Universidad de Buenos Aires, Buenos Aires, Argentina; 3 Institut de Biologie Structurale Jean-Pierre Ebel, CEA-CNRS-UJF, Grenoble, France; 4 Chimie Organique de Synthèse, Institut de Chimie B6A, Sart-Tilman. Université de Liège, Liège, Belgium; 5 Université de Paris-Sud 11 and CNRS, Institut de Biochimie et Biophysique Moléculaire et Cellulaire, Laboratoire des Enveloppes Bactériennes et Antibiotiques, UMR 8619, Orsay, France; Dartmouth Medical School, United States of America

## Abstract

To resist to β-lactam antibiotics Eubacteria either constitutively synthesize a β-lactamase or a low affinity penicillin-binding protein target, or induce its synthesis in response to the presence of antibiotic outside the cell. In *Bacillus licheniformis* and *Staphylococcus aureus*, a membrane-bound penicillin receptor (BlaR/MecR) detects the presence of β-lactam and launches a cytoplasmic signal leading to the inactivation of BlaI/MecI repressor, and the synthesis of a β-lactamase or a low affinity target. We identified a dipeptide, resulting from the peptidoglycan turnover and present in bacterial cytoplasm, which is able to directly bind to the BlaI/MecI repressor and to destabilize the BlaI/MecI-DNA complex. We propose a general model, in which the acylation of BlaR/MecR receptor and the cellular stress induced by the antibiotic, are both necessary to generate a cell wall-derived coactivator responsible for the expression of an inducible β-lactam-resistance factor. The new model proposed confirms and emphasizes the role of peptidoglycan degradation fragments in bacterial cell regulation.

## Author Summary

Beta-lactamases are enzymes produced by some bacteria and are responsible for their resistance to beta-lactam antibiotics like penicillin. Among these bacteria some of them possess a beta-lactamase that is only produced at a high level when a beta-lactam antibiotic is present outside the cell. This mechanism of regulation, named beta-lactamase induction, has been described both in Gram-negative and Gram-positive bacteria. In *Staphylococcus aureus* and *Bacillus licheniformis*, two Gram-positive bacteria, their beta-lactamase is induced by homologous proteins including a membrane-bound penicillin receptor, BlaR1, and a cytoplasmic DNA-binding protein, BlaI, acting as repressor. The first step in the induction mechanism is the acylation of the extracytoplasmic domain of BlaR receptor by the beta-lactam antibiotic concomitant with the activation of the peptidase activity of its cytoplasmic domain. The activated receptor launched a cytoplasmic signal leading to the inactivation of BlaI repressor. The nature of this cytoplasmic signal is not well understood. In our study, we identified that a dipeptide issued from the bacterial cell wall is able to inactivate the BlaI repressor. It is the first time that a cell wall fragment is shown to reenter in the cytoplasm of Gram-positive bacteria to regulate gene expression.

## Introduction

The introduction of penicillin, a β-lactam antibiotic, to treat bacterial infection, has drastically reduced the cases of human morbidity and mortality. However, a tight control of these bacterial pathogens has never been achieved and the use of β-lactam antibiotics appears to be linked to the selection and the spread of β-lactam resistant clinical isolates. In these strains, β-lactam antibiotic resistance can be obtained by one of the three following mechanisms: (i) inactivation of the β-lactam molecule by a specific hydrolase, the β-lactamase [1]–[3]; (ii) alteration of the β-lactam targets, the membrane-bound D,D-transpeptidases, that renders them insensitive to the action of the antibiotic [4]. These enzymes, which catalyze the final step of the bacterial cell wall biosynthesis, are inactivated by penicillin through acylation of their active serine by the antibiotic. For this reason, these enzymes are reported in the literature as Penicillin-Binding Proteins (PBPs) [3], [5] and (iii) prevention of β-lactams from reaching their targets. This mechanism of resistance is only found in Gram-negative bacteria and can be due to the alteration of porins and/or the presence of an efflux pump [6]–[8].

In Gram-negative bacteria, the presence of a β-lactamase, sometimes in synergy with decreased outer membrane permeability or efflux systems, is the main resistance factor [9] whereas a low-affinity PBP (resistant PBP) is the most frequent factor encountered in Gram-positive bacteria [3].

**Figure 1:**
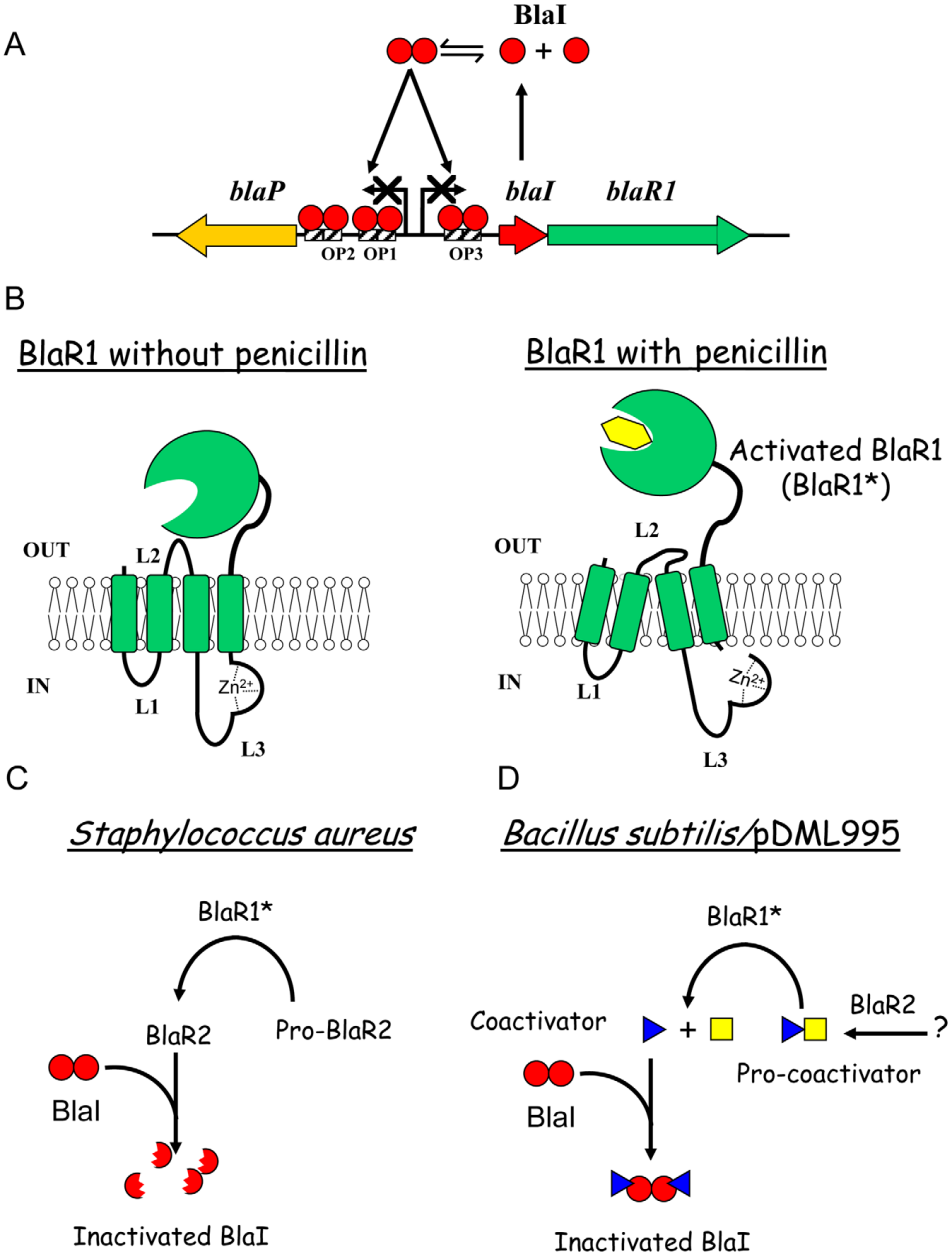
Beta-lactamase induction in *B. licheniformis* 749/I and *S. aureus*. (A) *Organization of bla divergon*: on the *B. licheniformis* 749/I chromosome, *blaP*, *blaI* and *blaR1* form a divergon; the transcription of *blaI* results in the biosynthesis of a dimeric BlaI repressor that binds to three operators (OP1, OP2 and OP3) located between the *blaP* and *blaIR1* operons, preventing *blaP*, *blaI* and *blaR1* transcriptions. A similar gene organization is present in *S. aureus*. (B) *Activation of BlaR/MecR membrane-bound penicillin receptor by a β-lactam*: BlaR/MecR is a membrane protein constituted by two domains: a N-terminal domain (BlaR-NTD) containing four transmembrane segments and an extracellular C-terminal domain (BlaR-CTD) able to bind penicillin; when a β-lactam is present outside the cell, BlaR-CTD is acylated and a signal is transmitted through transmembrane segments to the BlaR-NTD intracellular domain that leads to the proteolytic activation of its metalloprotease activity. (C and D) *BlaI inactivation*: In *S. aureus*, BlaI would be directly or indirectly cleaved by the activated metalloprotease, the resulting degraded repressor loses its ability to bind DNA and the expression of the resistance gene is launched. In BS995, BlaI inactivation could be the result of the presence of a coactivator.

In Eubacteria, β-lactamases can be either constitutively expressed or induced by a sub-lethal concentration of β-lactam antibiotic outside the cell [10]. The *Staphylococcus aureus* BlaZ and *Bacillus licheniformis* 749/I BlaP β-lactamases are under the control of at least three different gene products: BlaI, BlaR1 and BlaR2 homologous for both species. BlaI and BlaR1 act as a cytoplasmic repressor and a membrane-bound penicillin receptor, respectively [11]. The implication of a *blaR*2 gene has been deduced from genetic studies but this is not yet demonstrated [12], [13]. On the bacterial chromosome, *blaP/Z*, *blaR*1 and *blaI* are clustered in a divergon (*bla* divergon) in which *blaR*1 and *blaI* form an operon (Figure 1A). Similarly, two regulatory genes, *mecR1* and *mecI*, homologous to *blaR*1 and *blaI*, have been identified in *S. aureus* and are involved in the induction of the low affinity PBP2a (encoded by *mecA* gene). The two regulatory genes are organized in an operon and form, together with *mec*A, a divergon (*mec* divergon). In the induction of PBP2a, the MecI and MecR1 proteins have the same function as BlaI and BlaR1 [14]. In addition, sequence similarities between the promoter-operator regions of the *mecA* and *blaZ/P* divergons have been observed. Furthermore, the purified MecI and BlaI bind *mec/bla* operators [15], [16]. *In vivo*, the *mecR*1 operon regulates the PBP2a production in *S. aureus* [17] and if the staphylococcal MecI and BlaI repressors are interchangeable, the BlaR1/MecR1 penicillin receptors are not. Indeed, the complementation of a *mecR*1 mutation present in the *mec* divergon cannot be achieved by the *S. aureus blaR*1 [17].

In the absence of β-lactam antibiotic (further referred as the inducer), the BlaP/BlaZ β-lactamase synthesis and *blaIR*1 operon expression remain low thanks to the binding of the homodimeric BlaI repressor to its operator in the *bla* divergon (Figure 1A). BlaR1 acylation by the antibiotic launches a cytoplasmic, receptor-dependent signal that will lead to BlaI inactivation. Consequently, the derepression of the *blaZ/blaP* and *blaIR*1 genes increases the β-lactamase synthesis by the bacterial cell. In 2001, Zhang *et al* have proposed a mechanism explaining the β-lactamase induction in *S. aureus* [18]. In this model, the first consequence of the acylation of the BlaR1 sensor domain is the single point cleavage of its L3 cytoplasmic loop. This cleavage converts the putative inactive L3 metalloprotease domain into an active form (Figure 1B). Next, the activated L3 metalloprotease directly or indirectly inactivates BlaI by cleavage of the peptide bond linking the N101 and F102 residues (Figure 1C). The cleaved BlaI repressor exhibits a low affinity for its nucleic operator and no longer represses the transcription of *bla* divergon. In this model, BlaR2 could be activated by BlaR1 to cleave BlaI or involved in BlaR1 activation (Figure 1C). In the latter case, the activated BlaR1 would be directly responsible for the BlaI cleavage. Although this model can describe the fate of BlaI/MecI in *S. aureus*, the molecular details underlying the induction mechanism remain unclear. This aspect has been highlighted when the three dimensional structures of the staphylococcal BlaI and MecI proteins were obtained. Indeed, these structures showed that the site of cleavage was buried and inaccessible to the solvent [14], [19]. In *B. licheniformis* 749/I, the fate of BlaI during the BlaP induction is similar to that described for the staphylococcal repressor, except that in that organism, BlaI is completely degraded during the induction [12]. However, unexpectedly, in a *Bacillus subtilis* 168 strain carrying a plasmid harbouring the *B. licheniformis blaP-blaI-blaR*1 divergon (pDML995), the BlaP β-lactamase is induced in the *B. subtilis* genetic background and the BlaI repressor is inactivated without proteolysis [12], [20]. The ability of this recombinant *B. subtilis* 168/pDML995 (BS995) to induce the BlaP β-lactamase implies that an orthologous *blaR2* gene is present in the *B. subtilis* 168 genome and that the inactivation of BlaI could be the result of the presence of a coactivator produced independently of the presence of the *bla* divergon [12]. Furthermore, from kinetic studies of the BlaP induction, Duval *et al* [21] deduced that two conditions must be fulfilled to achieve β-lactamase induction in *B. licheniformis* 749/I: (i) BlaR1 must be activated by the β-lactam antibiotic and (ii) the antibiotic must generate an intracellular penicillin stress. All these results (obtained in *Bacillus*) and the buried cleavage site in the three-dimensional structures of the staphylococcal BlaI/MecI repressors suggest the presence of a molecule inactivating BlaI/MecI (Figure 1D). To probe this hypothesis, we have revisited the induction mechanism by searching for the presence of a coactivator.

## Results

### A cytoplasmic coactivator that inactivates BlaI is present in the cytoplasm of induced *B. subtilis*/pDML995 (BS995)

**Figure 2:**
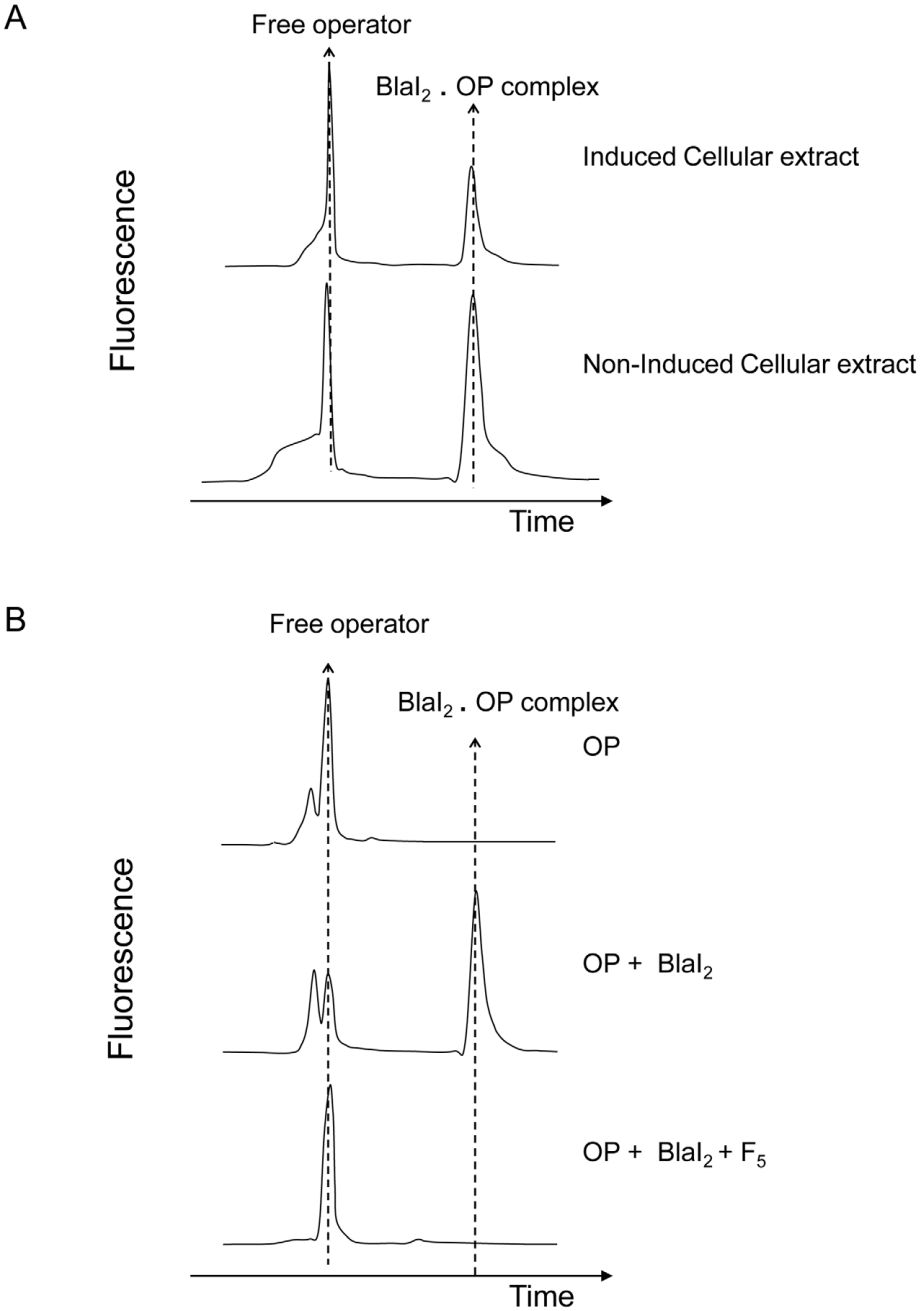
Fluorescent EMSA demonstrate the presence of a coactivator in induced BS995 cellular extracts. (A) Ability of ultrafiltrated non-induced and induced cellular extracts (molecular mass: ≤10 kDa) to destabilize the BlaI_2_.operator complex (BlaI_2_.OP). After peak integration, the ratio of bound OP/total OP is 0.32 and 0.57 for induced and non-induced cellular extracts, respectively. EMSA were carried out by using an ALF DNA sequencer. (B) Ability of the F5 fraction (molecular mass: 300–600 Da) to destabilize the BlaI_2_.OP complex. This fraction was obtained by fractionating an induced cellular extract on Sephadex G-25 (for details see Materials and Methods and Supplemental data). In presence of a compound present in F5 fraction the BlaI_2_.OP complex is dissociated (lower EMSA). Electropherograms showing free OP and BlaI_2_.OP complex are provided for control.

In a previous work, Filée *et al* have postulated the presence of a coactivator in the cytoplasm of induced BS995 cells [12]. To check this hypothesis, we have prepared small-scale soluble crude cellular extracts of non-induced and induced BS995 cells (for details see Materials and Methods). These extracts were ultrafiltrated on a 10 kDa cut-off membrane to eliminate high-molecular-mass macromolecules, and submitted to a fluorescent electrophoretic mobility shift assay (EMSA) [22]. As shown in figure 2A, only the partially purified induced cellular fraction is capable to destabilize the interaction between the dimeric *B. licheniformis* BlaI repressor and its nucleic operator (BlaI)_2_.OP. When the ultrafiltrated fraction of the induced cellular extract was incubated for 10 min at 100°C and resubmitted to EMSA, no heat effect was detected. The remaining heated fraction was further fractionated by ultrafiltration on a 5 kDa cut-off membrane and the resulting ultrafiltrated material retained its ability to disrupt the (BlaI)_2_.OP complex (data not shown). At this step, we concluded that a thermostable coactivator with a molecular mass lower than or equal to 5 kDa was present in the cytoplasm of induced BS995 cells, which was responsible for the inactivation of BlaI during the induction process.

To determine when the production of coactivator reaches its maximum during the induction process, small-scale soluble cellular extracts of induced BS995 cells were prepared every 15 min from 0 to 180 min after the addition of the inducer. By fluorescent EMSA, the production of coactivator reached a maximum level between 75 and 105 min after the addition of the inducer (Figure S1 in Text S1). This result is in agreement with the maximum rate of β-lactamase synthesis that is reached after 80–90 min in *B. licheniformis* 749/I or BS995 [21]. In the following experiments, it was assumed that the peak of coactivator production was reached 90 min after the addition of the inducer.

To characterize the coactivator, a large scale extract was prepared by inducing BS995 cells and harvesting 90 min after the addition of the inducer. This cellular extract was heat-treated, partially purified by ultrafiltration on a 10 kDa cut-off membrane and freeze-dried. The dried residue was then dissolved in 50 mM NH_4_HCO_3_ (pH 7.8) buffer and submitted to molecular sieving on a Sephadex G-25 column (1×100 cm) equilibrated in the same buffer. The chromatogram obtained by monitoring the eluate absorbance at 215 nm supplied eight major peaks (F_1_, …, F_8_, Figure S2 in Text S1) and the corresponding fractions were pooled and freeze-dried. Only the F_5_ peak showed an ability to destabilize the (BlaI)_2_.OP complex using the fluorescent EMSA technique (Figures 2B and S2 in Text S1). The estimated average molecular mass of the components in the F_5_ peak varied from 300 to 600 Da, an estimation obtained by comparing the average distribution constant (K_av_ = 0.62) of F_5_ fractions to those obtained for tryptic peptides [23] or for a small molecule such as 6-amino-penicillanic acid (216 Da, K_av_ = 0.66) [24]. At this point, we postulated that the coactivator generated by the penicillin-induction was a molecule with a molecular mass in the 300–600 Da range. Thus, the next question concerned the origin of this coactivator.

In their study of the BlaP induction of *B. licheniformis*, Duval *et al* [21] stated that two conditions must be fulfilled to allow induction, namely (i) the acylation of the BlaR receptor by the β-lactam antibiotic and (ii) a cellular stress probably due to the partial acylation of PBP1 by the β-lactam. The latter condition suggests that the PBP1 acylation could trigger a higher autolytic activity leading to an overproduction of peptidoglycan fragments outside the cell. These fragments could be transported into the bacterial cell by an unknown mechanism and one of them, modified by the activated BlaR1, could act as the coactivator. To investigate this hypothesis, various peptidoglycan fragments were assayed for their ability to destabilize the (BlaI)_2_.OP complex.

### The coactivator is a peptidoglycan fragment

**Figure 3:**
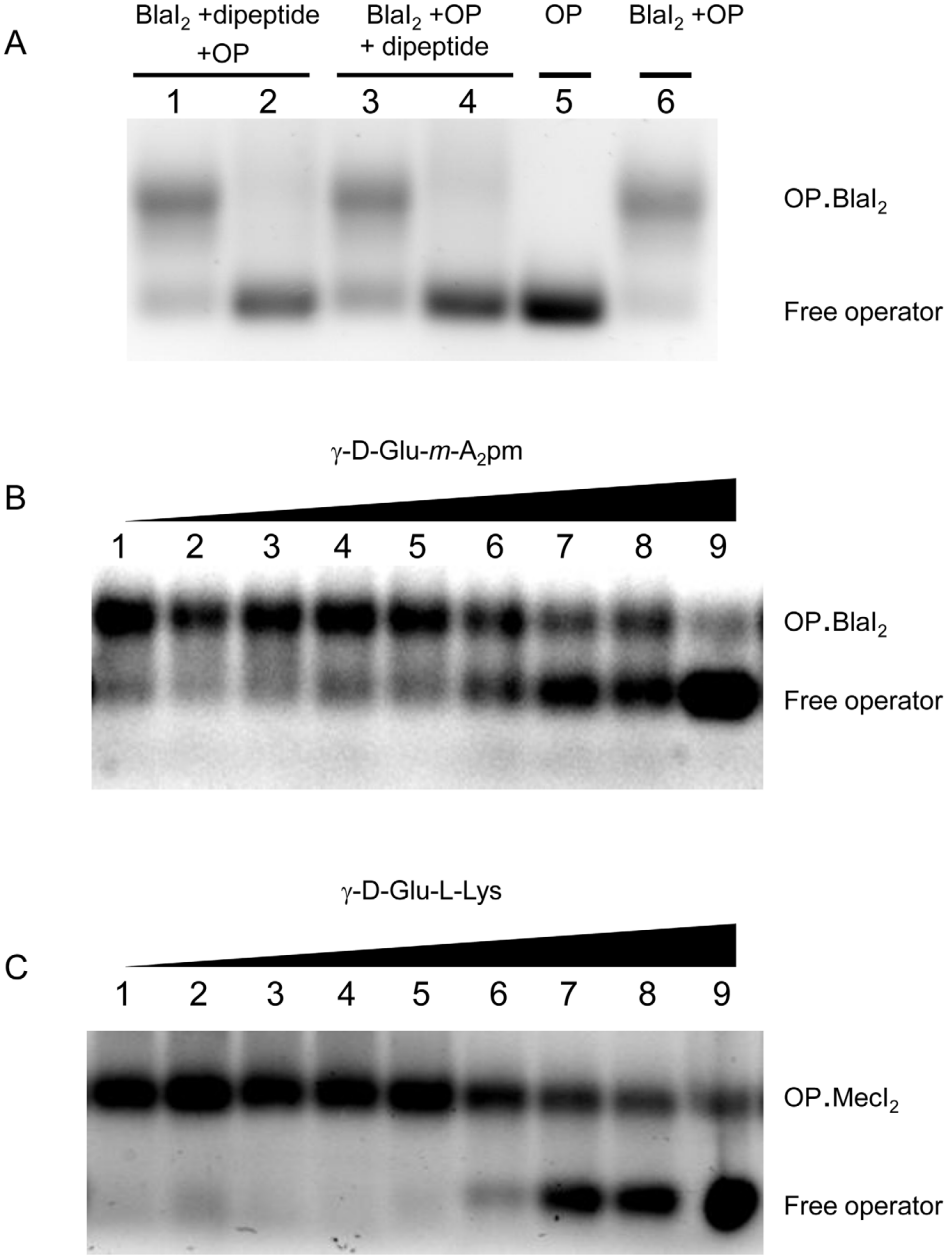
EMSA of BlaI/MecI.OP complex in presence of dipeptide. (A) Effect of γ-D-Glu-*m*-A_2_pm on BlaI_2_.OP complex. Two procedures were tested: either all compounds were added at the same time (lanes 1–2) or BlaI_2_.OP complex was formed prior to the addition of the dipeptide (lanes 3–4). For lanes 1, 2, 3, 4 and 6, 1 nmol BlaI and 50 pmol OP were used to form BlaI_2_.OP complex. For lanes 1 and 3, 10 nmol of γ-D-Glu-*m*-A_2_pm (dipeptide 1) were added, whereas 100 nmol were added in lanes 2 and 4. Lane 5: 50 pmol of OP. Total volume: 20 µl. (B) Titration of BlaI_2_.OP complex by the γ-D-Glu-*m*-A_2_pm dipeptide. After BlaI_2_.OP complex formation (1 nmol BlaI and 50 pmol OP), the dipeptide was added in increasing amounts: 10, 15, 20, 25, 30, 35, 40, 45 and 50 nmol, respectively (lanes 1–9). Total volume: 20 µl. (C) Titration of MecI_2_.OP complex by the γ-D-Glu-L-Lys dipeptide. After MecI_2_.OP complex formation (1 nmol MecI and 50 pmol OP), the dipeptide was added in increasing amounts: 10, 15, 20, 25, 30, 35, 40, 45 and 50 nmol, respectively (lanes 1–9). Total volume: 20 µl. EMSA were carried out on agarose gels. For details see Materials and Methods.

The various peptidoglycan fragments or related molecules listed in Table 1 were tested by fluorescent EMSA. Among these 20 compounds, only two peptides, γ-D-Glu-*m*-A_2_pm (dipeptide 1, where A_2_pm represents diaminopimelic acid) and γ-D-Glu-L-Lys (dipeptide 2), derived from *Bacillus subtilis* and *S. aureus* peptidoglycans, respectively, can dissociate the *B. licheniformis* (BlaI)_2_.OP complex. Both compounds tested on the homologous staphylococcal MecI repressor showed the same effect. To confirm the positive results obtained with the highly sensitive fluorescent EMSA, the experiments were repeated by using the less sensitive EMSA performed with an agarose gel as separation matrix. Under these conditions, higher concentrations of BlaI and operator could be used and more reproducible results were obtained. Dissociation of the (BlaI)_2_.OP complex was again observed and a titration of the repressor/DNA complexes by their respective dipeptides performed (Figure 3). From these data, the concentration of dipeptide allowing the displacement of 50% of bound BlaI/MecI to their nucleic operators was estimated at about 2 mM.

**Table 1:** Peptidoglycan-related molecules tested for their ability to disrupt BlaI/MecI repressor-operator complexes by EMSA (a).

*COMPOUND STRUCTURE*	*BlaI inactivation*	*MecI inactivation*	*Origin*
UDP-MurNAc-L-Ala-γ-D-Glu-*m*-A_2_pm-D-Ala-D-Ala(b)	no	no	[58]
MurNAc-L-Ala-γ-D-Glu-*m*-A_2_pm -D-Ala-D-Ala(b)	no	no	[59]
GlcNAc-MurNAc(anhydro)-L-Ala-γ-D-Glu-*m*-A_2_pm-D-Ala(b)	no	no	[60]
GlcNAc-MurNAc(anhydro)-L-Ala-γ-D-Glu-*m*-A_2_pm(b)	no	no	[60]
GlcNAc-MurNAc(anhydro)	no	no	[60]
L-Ala-γ-D-Glu-L-Lys-D-Ala(c)	no	no	This work
L-Ala- γ-D-Glu-*m*-A_2_pm(b)	no	no	This work
γ-D-Glu-L-Lys-D-Ala-D-Ala(c)	no	no	This work
γ-D-Glu-L-Lys-D-Ala(c)	no	no	This work
γ-D-Glu-L-Lys(c)	yes	yes	This work
γ-D-Glu-*m*-A_2_pm(b)	yes	yes	[59] and this work
D-Ala-D-Ala	no	no	This work
γ-D-Glu-Gly	no	no	Bachem
L-Glu	no	no	Bachem
L-Gln	no	no	Bachem
A_2_pm (racemic mixture of LL, DD and *meso* derivatives)	no	no	Sigma
L-Lys	no	no	Sigma
γ-L-Glu-L-Lys	no	no	This work
D-Gln-NH_2_	no	no	Bachem
D-Gln	no	no	Bachem
Ac_2_-L-Lys-D-Ala-D-Ala	no	no	[61]

(a) See Materials and Methods for more details.

(b) Peptidoglycan precursors or fragments derived from *E. coli*, *B. subtilis* or *B. licheniformis* cell walls.

(c) Peptidoglycan fragments derived from *S. aureus* cell wall.

MurNAc: *N*-acetyl-muramic acid; GlcNAc: *N*-acetyl-glucosamine.

At this step, it could be concluded that the molecule responsible for the inactivation of BlaI/MecI was a dipeptide derived from peptidoglycan.

### Identification of the coactivator present in the BS995 cellular extract

In order to establish whether dipeptide 1 was present in the active F_5_ fraction, we examined the sample elution pattern by RP-HPLC. When analyzed by HPLC on a C_18_ reverse-phase column in acidic condition (0.1% trifluoroacetic acid (TFA) in water (buffer A), dipeptide 1 was not retained by the hydrophobic matrix and eluted in the void volume of the column. To overcome this problem, dipeptide 1 was modified with 2,4,6-trinitrobenzene sulfonic acid (TNBS). Upon reaction with TNBS, primary amines form a highly chromogenic trinitrophenyl-derivative (TNP-), which is more hydrophobic and can be detected at 335 nm. As dipeptide 1 contains two free amino groups, two peaks corresponding to the mono- and di-TNP derivatives of this compound were observed, which were eluted at 35% and 55% of acetonitrile, respectively.

Next, in the text, only the results obtained for the fraction eluted at 35% of acetonitrile will be reported; the same conclusions are valid for the second fraction. Analysis of the TNBS-modified fraction by RP-HPLC showed that a peak corresponding to dipeptide 1 was present in the F_5_ fraction. This peak was specifically enriched by affinity chromatography when BlaI_2_.OP was used to trap the dipeptide 1 present in this fraction (see Figure S3 in Text S1). Furthermore, the enriched peak co-eluted with synthetic dipeptide 1 when the latter was added to the F5 fraction before TNBS modification. At this step, it was concluded that dipeptide 1 was effectively present in induced BS995 cells.

### The coactivator directly interacts with the C-terminal domain of BlaI/MecI

To point out a direct molecular interaction between the BlaI/MecI proteins and dipeptides 1 and 2, NMR saturation transfer difference (STD) experiments were carried out. STD is particularly well adapted to detect ligands that weakly bind to their protein receptors (dissociation constants, K_d_, in the mM range, [25]).

First, for STD experiments, a narrow frequency region of BlaI/MecI proton resonances where proton dipeptide resonances are absent was selectively saturated. Due to the large molecular mass of BlaI/MecI, spin diffusion is able to progressively propagate the saturation to other repressor protons, including the area of the putative binding pocket. In the case of complex formation between BlaI/MecI and one of the two dipeptides, the saturation will be also transferred to the bound dipeptide which in turn is rapidly exchanged from its bound form to its free form in solution. Since ligands are typically small molecules with long relaxation times, the saturation information is able to persist for a long time during which new previously unsaturated ligands can also bind with saturated BlaI/MecI. In this way, the population of saturated ligands in solution increases and the corresponding proton ligand resonances can be detected.

**Figure 4:**
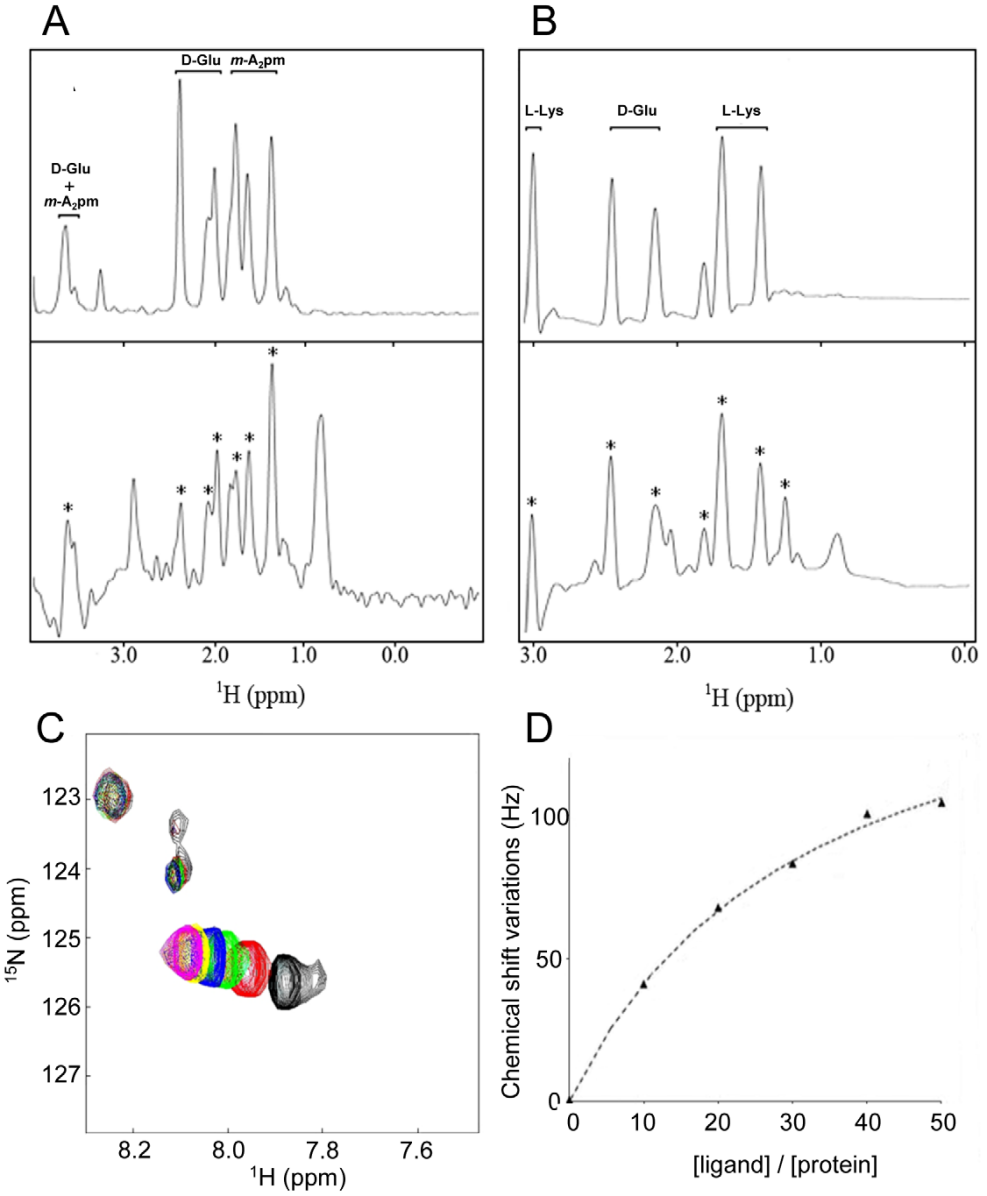
Characterization of the direct interaction between BlaI/MecI repressors and the dipeptide ligands using STD methods and chemical shift mapping by NMR. (A) Aliphatic region of ^1^H spectrum of γ-D-Glu-*m*-A_2_pm (top panel) and the STD experiments performed on the same peptide after the addition of BlaI repressor at a [Ligand]/[Protein] ratio of 50 (bottom panel). (B) Aliphatic region of the spectrum collected on γ-D-Glu-L-Lys fragments (top panel) and the STD experiments performed on the same peptide after the addition of the MecI repressor at a [Ligand]/[Protein] ratio of 50 (bottom panel). For (A) and (B), peaks labeled with a star correspond to dipeptide resonances that can be unambiguously assigned to a saturation transfer from the protein to the ligand, pointing out the direct interaction between the repressor and the peptidoglycan fragment. The few other peaks present in the STD experiment can be assigned to residual protein signals. They can be observed in STD experiments recorded on free protein samples. (C and D) ^15^N and ^1^H chemical shift variations observed on MecI in presence of different concentrations of γ-D-Glu-L-Lys dipeptide. The left panel (C) shows a region of Sofast-HMQC experiments performed on MecI repressor upon dipeptide addition. Spectrum of free MecI is plotted in black. Sofast-HMQCs displayed in different colors correspond to experiments collected on the same protein sample upon successive additions of dipeptide in order to obtain [Dipeptide]/[Protein] ratios of 10, 20, 30, 40 and 50 (plotted in red, green, blue, yellow and magenta, respectively). The right panel (D) shows the titration curve of the chemical shift perturbations observed on the ^15^N-^1^H correlation spectrum of MecI for different concentrations of γ-D-Glu-L-Lys dipeptide ([Ligand]/[Protein] ratios are displayed). ^15^N and ^1^H chemical shift variations were measured in Hz for peaks, added and finally divided by the number of selected peaks to obtain an average value. The final affinity constant was 8±2 mM. Error was estimated by Monte-Carlo simulations by assuming that a plateau is reached at a [Ligand]/[Protein] ratio of 50.

Thus, to highlight the interaction of the BlaI repressor with the γ-D-Glu-*m*-A_2_pm dipeptide and the association of the MecI protein with the γ-D-Glu-L-Lys dipeptide, STD experiments were performed on both systems (Figures 4 and S4 in Text S1). First, the STD experiment was performed on samples containing only the ligand or only the protein. Then, the spectra were recorded on mixtures including one of the proteins and its cognate dipeptide (Figure 4A–B). For BlaI and MecI, the presence of peptidoglycan fragment resonances in the STD experiment clearly demonstrated a saturation transfer from the protein to the dipeptide. This revealed a direct interaction between the *B. licheniformis* BlaI and the γ-D-Glu-*m*-A_2_pm dipeptide. Similarly, we observed an association between the *S. aureus* MecI and the γ-D-Glu-L-Lys fragment.

In addition, to investigate the effect of the binding of dipeptides 1 and 2 to the proteins, the complex formation was followed by recording ^15^N-^1^H heteronuclear correlation spectra of samples containing ^15^N BlaI or ^15^N MecI proteins in the absence or presence of dipeptide (Figure 4A). For both proteins, chemical shifts or line intensity variations were induced by the addition of an excess of dipeptide (BlaI/dipeptide: 1/50), confirming a specific interaction with the peptidoglycan fragments.

However, in both cases, the chemical shift perturbations concerned a limited set of protein resonances. Superimposition of the spectra previously obtained with the BlaI/MecI N-terminal domains [26] onto those obtained for full-length BlaI/MecI in presence of their coactivator highlighted that the N-terminal domains did not interact with the dipeptides. These results suggested that the coactivator-binding site was located in the repressor C-terminal domains. However, due to the small dispersion of the C-terminal resonances [26], a more specific localization of the interaction surface was not feasible.

### Thermodynamic characterization of the MecI/γ-D-Glu-L-Lys association

Isotopically enriched *S. aureus* ^15^N-MecI was titrated with unlabeled γ-D-Glu-L-Lys coactivator. After each addition of dipeptide, a two dimensional ^1^H-^15^N correlation spectrum of ^15^N-labeled protein was recorded and MecI•dipeptide concentrations were determined by following chemical shift variations detected on the C-terminal resonances of the repressor. The dissociation constant of MecI•dipeptide complex was determined by using the following scheme in which one dipeptide molecule is bound by the MecI monomer independently of the presence of MecI dimer.
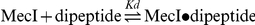
The fitting of the data obtained were in agreement with this scheme and the binding of dipeptide 2 with MecI was characterized by a K_d_ value of 8±2 mM (for more details see Materials and Methods). Following the same model, the K_d_ value obtained by EMSA titration was around 2 mM (Figure 3). In spite of the great stability of the MecI preparation during NMR experiments (stable for months at 4°C), the same preparation of MecI complexed by its dipeptide coactivator was more susceptible to proteolysis (Figure S5 in Text S1). This observation strengthened the explanation of MecI degradation by cytoplasmic proteases, when inactivated by the coactivator during induction.

### Evidence that cytoplasmic enzymes involved in peptidoglycan catabolism are linked to BlaP induction

The identification of the γ-D-Glu-*m*-A_2_pm as a cytoplasmic *B. licheniformis* BlaI inactivator suggests the presence of a protein machinery capable to generate this dipeptide from peptidoglycan catabolism. On the basis of this hypothesis, we have analyzed the *B. subtilis* protein database to find enzymes that could be involved in the hydrolysis of cytoplasmic peptidoglycan fragments. To do so, we searched the *B. subtilis* proteome for an ortholog of the *Escherichia coli* cytoplasmic L,D-carboxypeptidase (LdcA) which is involved in peptidoglycan recycling by releasing the terminal D-Ala from L-Ala-γ-D-Glu-*m*-A_2_pm-D-Ala [27]. In *B. subtilis* proteome, we identified the YkfA protein, a putative cytoplasmic L,D-carboxypeptidase (30% of sequence identity with *E. coli* LdcA). On the *B. subtilis* chromosome, the *ykfA* gene is included in an operon containing three other genes *ykfB*, *C* and *D* (*ykfABCD* operon). The functions of YkfB and YkfC are known. They are both cytoplasmic enzymes, with L-Ala-D/L-Glu racemase and γ-glutamyl-diaminoacid endopeptidase activities, respectively [28], [29]. The *ykfD* gene codes for a cytoplasmic protein homologous to the intracellular component of an oligopeptide ABC transporter [30]. The *ykfABCD* operon is also present in the genomes of other *Bacillus* species including that of *B. licheniformis*.

Accordingly, *B. subtilis* mutant strains carrying chromosomal deletions of each gene of the *ykf* operon were assayed for BlaP induction (for details see Text S1). The results obtained show that the inactivation of *ykfA* and *ykfB* negatively affected the BlaP induction by reducing the level of β-lactamase production by a factor of 2 and 1.5, respectively (Figure S7 in Text S1). The inactivation of *ykfC* does not have any effect on the induction mechanism whereas that of *ykfD* exhibits a delay of one hour in β-lactamase induction launching (Figures S7, C and D in Text S1). The lower β-lactamase production in the *ykfA* ^−^ and *ykfB* ^−^ mutants confirms that enzymes involved in peptidoglycan catabolism affect BlaP induction. However, the phenotype obtained for *ykfA* ^−^ and *ykfB* ^−^ mutants differs from that described for the BlaR2^−^ mutant, for which a non-inducible β-lactamase phenotype is reported. We sequenced and compared the complete *ykfABCD* operon from *B. licheniformis* WT and from BlaR2^−^ mutant and they are identical in both strains (data not shown). This result excludes the implication of the *ykfABCD* operon in the BlaR2^−^ mutant phenotype.

## Discussion

Within the limits of the compounds listed in Table 1, the molecule able to inactivate BlaI/MecI repressors is a dipeptide consisting of a D-glutamic acid residue linked to a diamino acid (L-Lys or *m*-A_2_pm) via a gamma-glutamyl peptide bond. This dipeptide is a specific fragment derived from the cross-linking peptide present in the peptidoglycan of most Gram-negative and Gram-positive bacteria. In *B. subtilis* and *B. licheniformis*, the MurNAc residue is substituted by the L-Ala-γ-D-Glu-*m*-A_2_pm-D-Ala tetrapeptide and cross-linking of the glycan chains occurs between the carboxyl group of the D-Ala of one chain and the free amino group of *m*-A_2_pm on a flanking chain [31]. In *S. aureus* L-Lys replaces *m*-A_2_pm in the peptide chain, and the cross-linking between two adjacent tetrapeptides is achieved *via* an additional penta-glycine bridge (L-Ala-γ-D-Glu-L-Lys[-ε-(Gly)_5_]-D-Ala [32].

### Origin of the dipeptide

An important question that remains to be answered is how the dipeptide is generated in the cytoplasm. It is well established that, in all Eubacteria, the peptidoglycan is continuously synthesized and degraded during cell growth [33]. This phenomenon is designated as the cell wall turnover and the degrading enzymes involved in this turnover are the autolytic enzymes. In *Enterobacteriaceae*, the peptidoglycan fragments generated by this catabolic activity are transported into the cytoplasm and the L-Ala-γ-D-Glu-*m*-A_2_pm tripeptide released during this process is efficiently recycled, *i.e.* reused for *de novo* peptidoglycan synthesis [33], [34]. In contrast, in Gram-positive bacteria, it has not been shown that the cell wall fragments generated by the autolytic enzymes are re-injected into the peptidoglycan biosynthesis pathway. But the alleged lack of peptidoglycan recycling in the latter group does not exclude that these fragments could be degraded and reused to produce other molecules than peptidoglycan precursors. Figure S6 in Text S1 summarizes the cell wall hydrolases that are known to cleave the *B. subtilis/B. licheniformis* peptidoglycan. Among these enzymatic activities, only the peptidase that cleaves the peptide bond between L-Ala and D-Glu has not been described for any eubacteria to date. In this way, the final product of all these catabolic activities is the dipeptide L-Ala-D-Glu. However, L-Ala-D-Glu can be epimerized to L-Ala-L-Glu by YkfB cytoplasmic epimerase (an activity also found in all *Enterobacteriaceae*) and the hydrolysis of the latter dipeptide could occur by known dipeptidases. In *B. subtilis*, the presence of YkfB and YkfC cytoplasmic enzymes, able to cleave peptides issued from peptidoglycan, suggests that these substrates generated by peptidoglycan hydrolases can be present in the *B. subtilis* cytoplasm.

During the BlaZ/BlaP/MecA induction process, the easy way to explain the formation of the dipeptide coactivator is to hypothesize that the activated BlaR would generate the dipeptide from L-Ala-γ-D-Glu-*m*-A_2_pm tripeptide. In this case, BlaR would act as an L-Ala-aminopeptidase. This is plausible because a His-Glu-X-X-His motif characteristic of neutral zinc peptidases is detected within the BlaR/MecR L3 loops. Moreover, in the neutral zinc peptidases super-family, the M1 subclass is composed of eukaryotic and prokaryotic proteins that exhibit an aminopeptidase activity [35], [36]. Besides, specific BlaR mutants in this neutral zinc-binding motif are unable to induce the synthesis of BlaP (B. Joris, unpublished results), reinforcing the hypothesis that BlaR could generate the dipeptide that inactivates the repressor. In the same way, we propose that the *Bacillus* pro-coactivator postulated by Filée *et al* [12] is the L-Ala-γ-D-Glu-*m*-A_2_pm tripeptide. The same authors have also suggested that the product of the unknown *B. licheniformis blaR*2 gene should be involved in the production of the pro-coactivator. As we have demonstrated that the γ-D-Glu-*m*-A_2_pm dipeptide acts as a BlaI coactivator, the *blaR*2 gene product could be part of the *B. licheniformis* cell wall hydrolases (autolytic system). This is in agreement with the results obtained by Duval *et al* [21], who showed that a penicillin stress, in addition to the BlaR acylation by the β-lactam, is necessary for the β-lactamase induction in *B. licheniformis*. This penicillin stress would stimulate the autolytic system, increasing the cell wall breakdown, thus explaining the high level of pro-coactivator generated in the presence of the inducer.

The best enzyme candidate to generate the pro-coactivator from L-Ala-γ-D-Glu-*m*-A_2_pm-D-Ala tetrapeptide is YkfA that could act as a L,D-carboxypeptidase. The *ykfA* inactivation does not abolish the induction phenomenon but leads to a decrease of β-lactamase induction. The impairment of β-lactamase induction in *ykfA* ^−^ mutant can be explained by the decrease of intracellular pro-coactivator concentration resulting in a lower concentration of coactivator available for BlaI inactivation. The unexpected negative effect of *ykfB* inactivation, for which the L-Ala-D-Glu dipeptide concentration should increase in the cytoplasm, can be explained by the fact that this dipeptide would act as a competitive inhibitor of the BlaR aminopeptidase activity. The expected phenotype for *ykfC* ^−^ mutant should be either a more or an unaffected inducible phenotype. Indeed, this last effect is observed. This phenotype could be explained by the inactivation of γ-glutamyl-diaminoacid endopeptidase activity and the subsequent accumulation of the BlaR substrate in the cytoplasm. The delay in β-lactamase induction for *ykfD* ^−^ mutant is in agreement with the inactivation of a protein involved in a specific metabolite transport. Notably, if YkfD is a part of an ABC transporter specific for peptidoglycan fragments generated outside the cell, its inactivation would generate the depletion of these fragments in the cytoplasm. The observation of a delay in β-lactamase induction suggests an alternative transport mechanism for these fragments taken up by a less efficient ABC transporter. This fact would explain why the pro-coactivator accumulation needed to launch BlaP induction takes more time to be reached.

The *ykfABCD* operon is not found in the *S. aureus* genomes. This could be explained by the fact that in the *S. aureus* peptidoglycan the cross-linking peptide is different. Nevertheless, *S. aureus* possesses all the enzyme activities necessary to cleave its peptidoglycan, including the glycyl-glycine endopeptidase LytM enzyme [37], [38].

### Mechanism of induction

**Figure 5:**
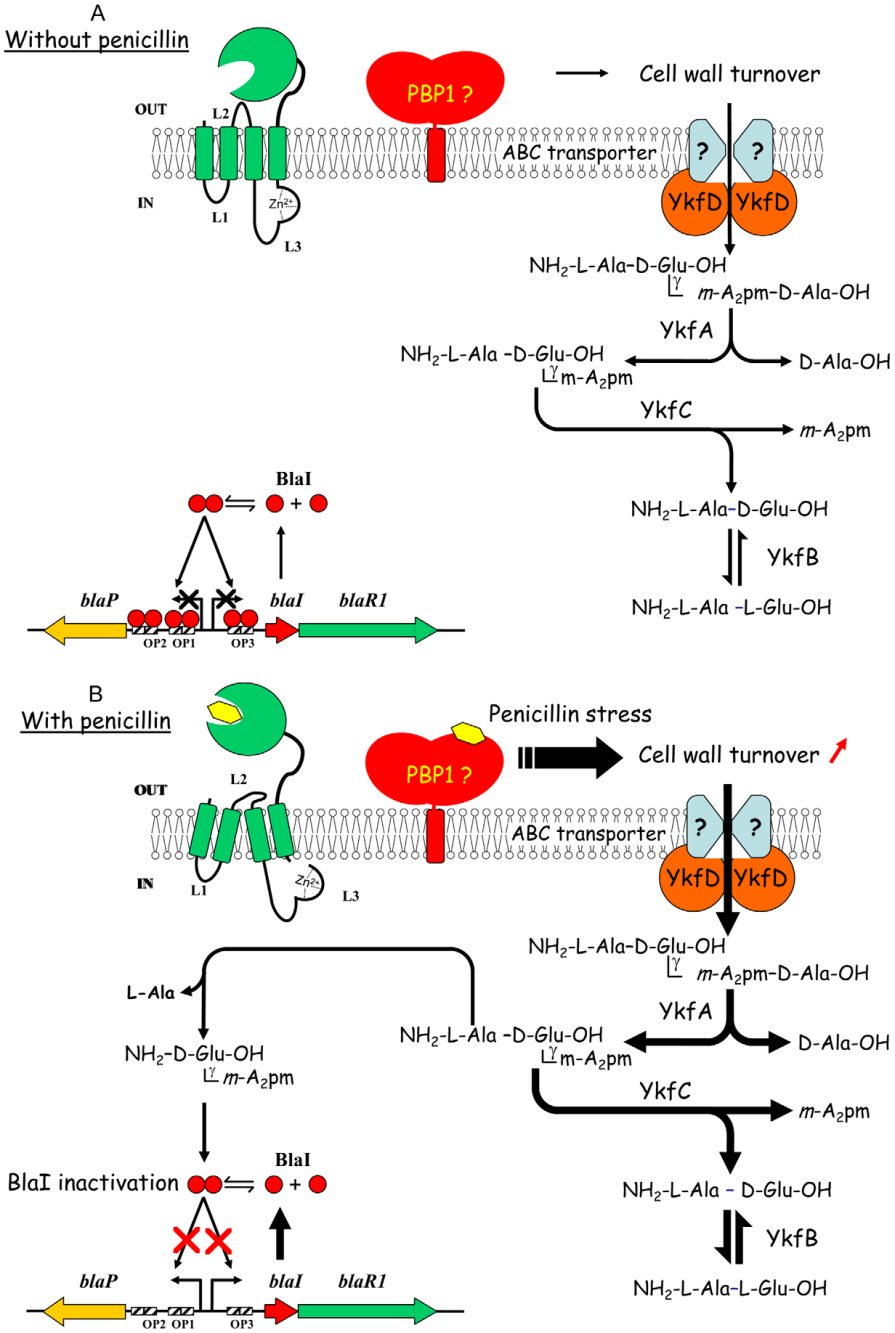
New model proposed for β-lactamase induction in *B. licheniformis* 749/I. (A) Without penicillin outside the cell, the BlaR penicillin receptor is not activated, BlaI dimer interacts with its nucleic operators (OP1, OP2 and OP3) and BlaP β-lactamase expression is maintained at a low level. PBP1 involved in the latest step of peptidoglycan biosynthesis is fully active and indirectly involved in cell wall turnover. Indeed, the incorporation of new glycan chains in the preexisting peptidoglycan network requires its partial hydrolysis. Peptidoglycan fragments generated by cell wall turnover are reused by *B. licheniformis* 749/I cells as source of carbon or nitrogen. The cytoplasmic YkfA, B, C and D proteins are probably part of a catabolic network involved in the use of peptidoglycan fragments generated by cell wall turnover. For more details see the text. (B) In presence of a concentration of penicillin such that the BlaR receptor and PBP1 are fully and partially acylated, respectively, two signals are generated into the bacterial cell. Partial PBP1 inactivation would generate a penicillin stress that would increase the cell wall turnover and the accumulation of peptidoglycan fragments in the cytoplasm. The penicillin-activated BlaR receptor would hydrolyze the L-Ala-γ-D-Glu-*m*-A_2_pm tripeptide, resulting from the activity of YkfA, to generate the γ-D-Glu-*m*-A_2_pm dipeptide. In this scheme, the tripeptide and the dipeptide are the pro-coactivator linked to *bla*R2 locus and the coactivator capable to inactivate the BlaI repressor, respectively. The binding of the coactivator to BlaI leads to the inactivation and subsequent release of the repressor in the cytoplasm where it can be hydrolyzed by cytoplasmic proteases.

The new model we propose, which is summarized in Figure 5, combines the integration of two signals generated by the action of the β-lactam inducer: BlaR1 activation and a cellular penicillin stress. The triggering event for the generation of these two signals is the acylation of BlaR1 and of one or several PBP(s) by the inducer. The acylation of PBPs results in their inactivation and leads to the perturbation of the cell wall biosynthesis, thereby provoking the anarchic activity of the autolytic enzymes that ultimately results in the accumulation of the cell wall fragments in the cytoplasm. The acylation of the BlaR1 C-terminal sensor domain by the inducer results in the rearrangement of its transmembrane segments and the activation of L3 cytoplasmic loop by self-proteolysis or proteolysis by a cytoplasmic protease. The activated L3 loop would act as an aminopeptidase to generate the γ-D-Glu-*m*-A_2_pm or γ-D-Glu-L-Lys coactivators from the corresponding tripeptides. The binding of the coactivator to BlaI/MecI would induce a conformational change that would make them unable to bind their DNA operators and to trigger the expression of BlaZ/BlaP/MecA. In the cytoplasm, the inactivated repressor would be hydrolyzed in the case of *S. aureus* and *B. licheniformis* by cytoplasmic proteases whose action would be facilitated by the binding of the coactivator, as observed during our NMR experiments with MecI.

In the new model proposed for BlaZ/BlaP/MecA induction, the inactivation of the homologous BlaI/MecI repressors is achieved by the same mechanism. It combines all the observations and deductions found in the literature for MecA/BlaZ/BlaP induction in *S. aureus*, *B. licheniformis* and *B. subtilis*. In the case of *B. subtilis*, the coactivator-repressor complex is insensitive to cytoplasmic proteases [12]. Furthermore, the BlaZ/BlaP/MecA expression is the consequence of the integration of multiple signals: BlaR1 acylation, PBP(s) inactivation, and cellular stress due to the presence of the inducer leading to the triggering of autolytic enzymes. The integration of all these ON inputs drives the output of a new AND gate regulation involving a peptidoglycan fragment [39].

In conclusion, our observations emphasize the muropeptide catabolic pathway present in *B. subtilis* [40], [41] and the role of peptidoglycan fragments in the signalling of β-lactam antibiotic stress response. It adds a new dimension to the emerging multiple roles of bacterial cell wall fragments in a surprisingly wide range of important biological phenomena. Indeed, different pathways are now known to be triggered by peptidoglycan fragments such as germination of *B. subtilis* dormant spores [42], induction of the AmpC β-lactamase in Gram-negative bacteria [43] and human innate immunity response involving Nod and peptidoglycan recognition proteins (PGRPs) [44], [45]. On the other hand, *B. subtilis* cell envelope stress responses include regulons controlled by σ^M^ elicited by antibiotics, σ^W^, σ^B^ and several two-component regulating systems [46]. In all these responses, the molecular effector signalling the cell envelope stress is missing. Could a peptidoglycan fragment be the general molecule controlling these regulons in *B. subtilis* and more generally in all Eubacteria? If so, this study on β-lactamase induction opens new ways for investigation and elucidation of bacterial cell regulation mechanisms.

## Materials and Methods

### Bacterial strains and plasmids

*Bacillus subtilis* 168 (ATCC 23857) was used as the host for pDML995 plasmid, a derivative of the *Bacillus/Escherichia coli* shuttle vector pMK4 [47] carrying the wild type *B. licheniformis* 749/I *blaP-blaIR*1 divergon, since this last strain is not transformable, preventing its easy genetic manipulation. *B. subtilis* 168 was transformed as described previously [48].

Luria-Bertani (LB) was used as liquid or solid (1.5% agar supplemented) medium for growing cells. Cultures were incubated at 37°C, with continuous shaking (250 rpm, Innova Shaker, New Brunswick Scientific Co.). Recombinant *B. subtilis* were selected with 7 µg/ml chloramphenicol.

The plasmid BlaIWTHis was constructed as described [49].

The plasmid MecIWTHis was constructed as follows: *mecI* from *Staphylococcus aureus* ATCC 43300 was amplified by PCR using primers mecIUP: 5′-GAG**CATATG**GATAATAAAA CGTATGAAATATCATC-3′ and mecIDW: 5′-**CTCGAG**TTTATTCAATATATTTCTCAATTCTTCTA-3′ (*Nde*I and *Xho*I recognition sequences respectively are in bold in the primer sequences). The 410 bp amplicon obtained was purified from agarose gel (PCR DNA and Gel Band Purification Kit, GE Healthcare) and cloned into pCR4.TOPO plasmid (Invitrogen), yielding pCRmecIHis. The latter plasmid was replicated in *E. coli* TOP10, purified, and both strands of the cloned fragment were verified by sequencing. The pCRmecIHis was digested by *Nde*I and *Xho*I (Promega, Madison) and the fragment cloned into *Nde*I and *Xho*I predigested pET22b (plasmid mecIHispET22b). In-frame cloning was verified by restriction digestion and sequencing. Six additional histidine residues at the C-terminal end of MecI were introduced in this protein by in-frame fusion of 3′ end of *mecI* and the pET22b.

Restriction endonucleases were purchased from Promega, Madison. Oligonucleotides were obtained from Eurogentec, Belgium. LA *Taq* Polymerase was provided by Takara Bio Inc. Routine DNA manipulations were performed as described by Sambrook and Russel [50]. DNA sequencing was performed by the dideoxy chain termination method using an ALFexpress sequencer (GE Healthcare Life Sciences).

### Overexpression and purification of MecI and BlaI

BlaI-His and MecI-His were, respectively, produced and purified as described previously [49], [51]. When proteins were overproduced for further RMN analysis, the same purification strategy was followed, but cells were grown in M9 medium [26] prepared with ^15^NH_4_Cl.

### Cellular extract preparation

*B. subtilis* 168 freshly transformed with plasmid pDML995 (BS995) was grown overnight in a small volume of LB supplemented with 7 µg/ml of chloramphenicol. The pre-culture was then diluted 1∶50 (v∶v) and incubated until an A*_600 nm_* of 0.6–0.8 was reached, when 2.5 µg/ml of the inducer cephalosporin C (final concentration) were added. Samples were taken at different times (depending on ulterior experiments) and centrifuged. The β-lactamase activity was determined in the supernatant by monitoring the hydrolysis of 100 µM nitrocefin. The cellular pellet was treated as follows:

- Small scale: cells were collected in DNA binding buffer [10 mM NaHPO_4_, pH 7.5, 50 mM KCl, 5% glycerol, 50 µg/ml BSA protease and nuclease free (Sigma Chemical Company)] and sonicated (3 bursts, 30 sec, pulse mode). The soluble cellular extract was recovered by centrifugation (2 min, 13000× *g* in a bench top microcentrifuge) and poured onto a 500 µl Vivaspin 10 kD protein concentrator (Sartorius). The filtrate, containing molecules smaller than the concentrator cut-off, was heated at 100°C for 10 min. The final protein concentration in this preparation was determined by the BCA protein assay kit (Pierce). If needed, an additional fractionation of filtrate was carried out by using a Vivaspin 5 kD protein concentrator (Sartorius).
- Scaling up: cells from a 2 liter culture, induced for 85–95 min, were collected in DNA binding buffer and disrupted by 3 passages through a French Press (SLM instruments, Urbana, IL). The soluble cellular extract was recovered by centrifugation at 19000 rpm for 30 min in a Sorvall RC5 centrifuge (SS34 rotor) and filtered through a 20 ml Vivaspin 10 kD protein concentrator following the manufacturer instructions (Sartorius). The filtrate was heated at 100°C for 10 min and then centrifuged at 19000 rpm for 40 min. This treated cellular extract was then freeze-dried and resuspended in 1 ml of water for further utilization.

### Electrophoretic mobility shift assay (EMSA)

Two methods were used:

- By using a 0.5 µM OP1 fluorescent double strand oligonucleotide: (5′-Cy5-GCATTTAAATCTTACATATGTAATACTTTC-3′) and 13.5 µM BlaI or MecI. They were allowed to form a complex in DNA binding buffer, for 150 min. Then, 250 µg of proteins coming from cellular extracts or different concentrations of peptides, amino acids or cell wall derivates were added in a final volume of 10 µl. The mixture was incubated overnight at 4°C and for an additional hour at 30°C. The band shift assay was carried out using an ALF express DNA sequencer [12], [14].
- By using the 2.5 µM double strand oligonucleotide and BlaI or MecI (50 µM). The complex was allowed to form in DNA binding buffer for 150 min at 30°C and its formation was checked by agarose gel electrophoresis. Then, different concentrations of dipeptides (from 10 to 50 fold the concentration of BlaI or MecI) were added to the complex and incubated overnight at 4°C and for 60 min at 30°C. Five µl of glycerol were added to the reaction mixtures before loading on a 1% (w∶v) agarose gel where bound and unbound oligonucleotides were separated by conventional gel electrophoresis. DNA was visualized by ethidium bromide staining.

### Source of putative coactivators

The sources and the structures of the compounds tested in this study as putative coactivators are listed in Table 1.

The dipeptide γ-D-Glu-*m*-A_2_pm was generated by treatment of the tripeptide L-Ala-γ-D-Glu-*m*-A_2_pm with aminopeptidase M (Roche Applied Science). The reaction mixture (100 µl) containing 50 mM sodium phosphate buffer pH 7.6, 1 mM tripeptide and aminopeptidase M (200 units) was incubated for 24 h at 37°C. The complete conversion of the tripeptide in Ala and dipeptide was confirmed by analysis of an aliquot with the aminoacid analyzer (Hitachi L8800, Science Tec). The dipeptide was then purified by HPLC on a Nucleosil 5C_18_ column (4.6×250 mm) using elution with 0.1% trifluoroacetic acid at a flow rate of 0.6 ml/min. Detection was performed at λ = 215 nm. Dipeptides 1 and 2 were also obtained by direct synthesis (Nathalie Teller, PhD Thesis, unpublished data).

The purity of the final products obtained for this study was verified by mass spectrometry and NMR spectroscopy (1D ^1^H experiment).

### NMR samples

The ^15^N BlaI sample concentration was 0.3 mM in a 75 mM NaH_2_PO_4_/Na_2_HPO_4_ buffer pH 7.6, containing 300 mM KCl in ^1^H_2_O∶^2^H_2_O 90∶10 (v∶v). To study the interaction, 94 µl of a sample of γ-D-Glu-*m*-A_2_pm was prepared at 69.5 mM in the previous buffer. The pH was adjusted to 7.6 before addition of the ligand in the ^15^N BlaI sample. The final protein∶ligand ratio obtained was 1∶50.

For the ^15^N MecI repressor, the sample was prepared at a concentration of 0.25 mM in 50 mM NaH_2_PO_4_/Na_2_HPO_4_ buffer, pH 7.6, containing 500 mM NaCl, 1 mM NaN_3_ in ^1^H_2_O∶^2^H_2_O 90∶10 (v∶v). The dipeptide γ-D-Glu-L-Lys, involved in the complex formation, was dissolved at a concentration of 100 mM in a buffer identical to the one used for the ^15^N MecI sample.

### STD experiments

Saturation Transfer Difference (STD) experiments [52], [53] were acquired at 25°C on a 600 MHz Varian Inova spectrometer equipped with a penta (^1^H/^2^H/^13^C/^15^N/^31^P) resonance probe with shielded z-gradients. In the sequence, saturation of a narrow band was achieved by a long pulse of 2.5 s at field strength of 48 Hz and was preceded by a supplementary recovery time of 1 s. The on-resonance irradiation of the protein was applied at a chemical shift of 0.8 ppm and 0.48 ppm for MecI and BlaI repressors, respectively. Using samples containing only the ligand or only the protein, the frequency of the selective saturation can be adjusted to improve the saturation transfer to the rest of the protein without affecting the peptide resonances. Off-resonance irradiation was set to −11.6 ppm, where no protein signals were present. The difference of the spectra recorded with on-resonance (I_sat_) and off-resonance (I_ref_) saturation provides the STD response:
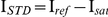
The spectra were substracted internally *via* phase cycling after every scan to minimize artifacts arising from spectrometer instabilities. For a target with a very large molecular mass, transverse relaxation is fast and permits to exclusively detect the signal corresponding to the free form of the substrate. In the case of the BlaI and MecI systems, a supplementary transversal relaxation filter was added to improve the cancellation of the protein signal. Based on differences of transversal relaxation rates between small and large molecular mass molecules, a spinlock was applied using a MLEV-16 [54] phase cycling at a field strength of 4000 Hz. The addition of such a pulse drastically reduces the protein signals without an important decrease of the signal of the free ligand. The pulse duration was adjusted to 200 ms in order to optimize the protein signal cancellation. Water suppression was finally achieved at the end of the sequence using a WATERGATE block [55]. Spectral signatures of the two dipeptides were obtained by recording one-dimensional NMR spectra of the dipeptides in absence of protein (Figure 4A–B). Peak identification was allowed thanks to two-dimensional TOCSY spectra collected on the free ligands in solution (data not shown).

### Heteronuclear experiments

^1^H-^15^N heteronuclear correlation experiments were measured on a 800 MHz Varian Inova spectrometer equipped with a triple (^1^H/^13^C/^15^N) cryogenic probe with shielded z-gradient. Regular HSQC spectra were recorded in total experimental times of 10 h with a spectral resolution of 17 Hz and 10 Hz in the direct and indirect dimensions, respectively. For sensibility improvement, SOFAST-HMQC sequence [56] was used with a 120 degree selective excitation of 2.25 ms duration. Concomitantly, a polychromatic PC9 shape, centered at 8.5 ppm and covering a bandwidth of 4 ppm, was required. Selective inversion was achieved using r-SNOB shape centered at 8 ppm and covering a bandwidth of 4 ppm. Spectral resolution was set to 43 Hz and 25 Hz in the indirect and direct dimensions, respectively. Recovery time was set to 0.3 s for a total experimental time of 17 h and 1 h in the case of experiments recorded for titration.

### Dissociation constant determination

As described previously, increasing amounts of the peptidoglycan fragment (γ-D-Glu-L-Lys) were successively added to the protein sample (*S. aureus* MecI). SOFAST-HMQC experiments, with an experimental time of 1 h, were recorded on a 600 MHz spectrometer after each addition of a small volume (10 µl) of a concentrated solution of dipeptide (100 mM). Thus, dilution was limited thanks to the availability of large quantities of the chemically synthesized ligand. Five spectra were collected upon dipeptide addition. Chemical shift variations measured indicated that the interaction partners were in fast exchange regarding the NMR time scale (Figure 4C). Only well-resolved peaks with a significant signal to noise ratio were considered in the fitting process. Standard second order polynomial expression was required to extract the affinity constant value (Figure 4D). Collected data were analyzed assuming that the detected chemical shift modification is a weighted average between the two extremes corresponding to the free (Δδ = 0) and the bound states (Δδ = Δδ_max_) [57]. A statistical analysis using Monte-Carlo simulations was performed to estimate the uncertainty of the processed data. Fit process and curve visualization were completed owing to the xmgrace software (http://plasma-gate.weizmann.ac.il/Grace/).

## Supporting Information

Text S1 **Supplemental data.**
(DOCX)
