# Supplementary material for: A Peptidoglycan Fragment Triggers β-lactam Resistance in Bacillus licheniformis
Source: PLoS Pathog. 2012 Mar 15;8(3):e1002571. doi: 10.1371/journal.ppat.1002571 (PMC3305447; doi:10.1371/journal.ppat.1002571)
Supplement: Text S1 — Supplemental data. (DOCX) [file ppat.1002571.s001.docx]

**SUPPLEMENTAL DATA**

**Figure S1: Time-course of co-activator production after induction with Cephalosporin C** BS995 induced cellular extracts (ICE) were prepared from samples withdrawn at 15 min intervals after the addition of the inducer (2.5 µg/ml cephalosporin C) as described in Experimental Procedures. 250 µg of proteins coming from each withdrawal were then added to a preformed BlaI_2_.OP complex (0.5 µM and 13.5 µM of OP and BlaI, respectively). Mixtures were incubated overnight at 4°C and thereafter for an additional hour at 30°C. The band shift assay was carried out with an ALF express DNA sequencer as described in Experimental Procedures. Free- and bound-OP represent the operator and the repressor-operator complex, respectively. The coactivator activity was estimated as the ratio of bound OP *versus* the sum of free and bound operator. The higher coactivator activities were obtained between 75 and 105 min after induction with Cephalosporin C.

NICE: non-induced cell extract.

**
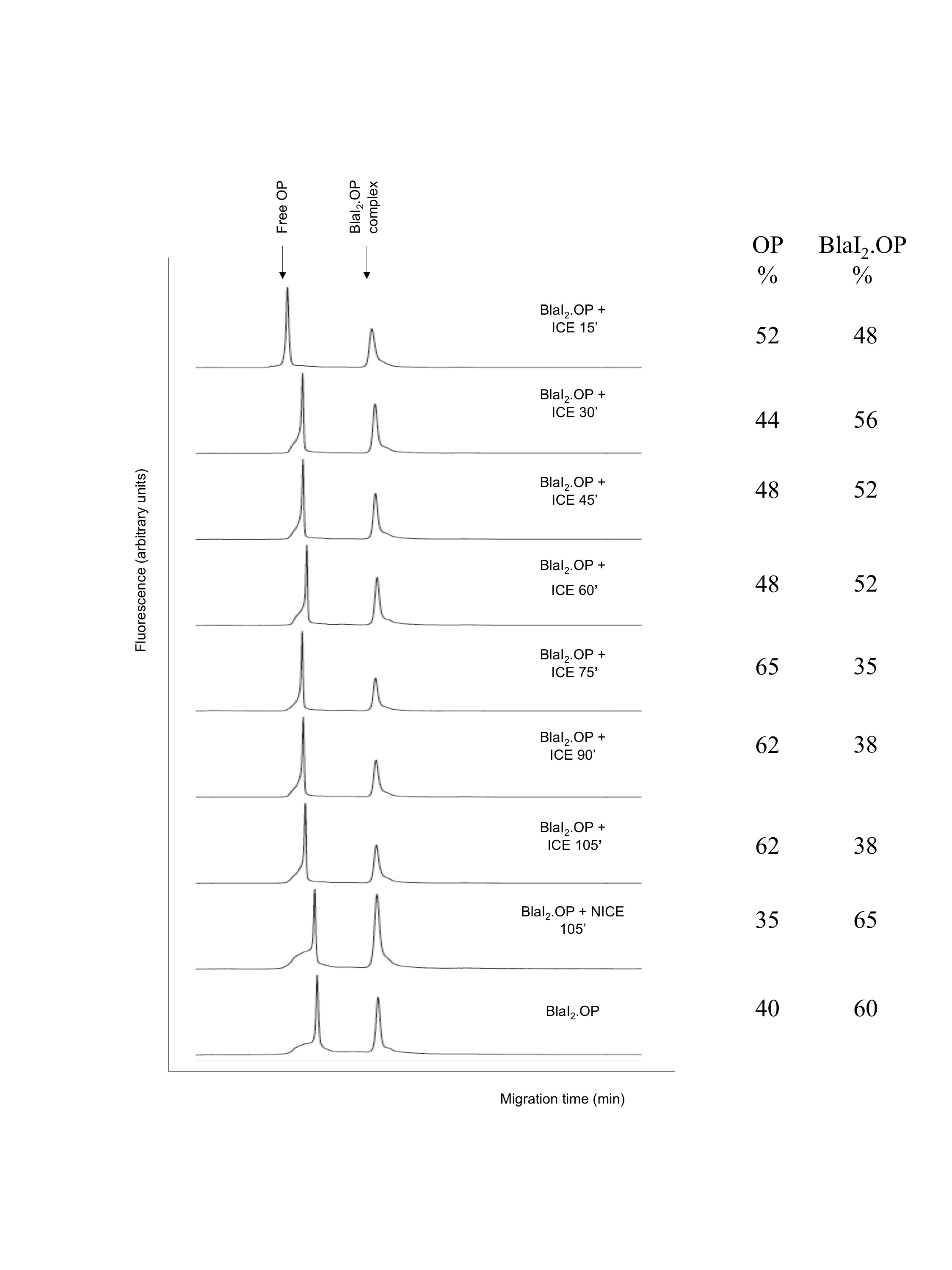
**

**Figure S2: Fractionation of large scale-induced cellular extract by molecular sieving.**

A cellular extract was obtained from a 2 l culture as described in experimental procedures. One milliliter of 50mM NH_4_HCO_3_ pH 7.8 (Buffer B) was added to the freeze-dried sample and then the sample was loaded onto a Sephadex G25 column (1 x 100 cm) equilibrated in buffer B**.** Elution of the sample was performed in the same buffer at 12 ml/h. 2 ml fractions were collected.

(A) Elution profile obtained by measuring the absorbance at 215 nm. Eight major peaks were obtained (F_1_ to F_8_). Fractions corresponding to the different peaks were pooled, freeze-dried and resuspended in 100 µl of water.

(B) Detection of coactivator activity in different fractions**.** An aliquot (4 µl) of each peak was tested by fluorescent EMSA for their ability to destabilize BlaI_2_-OP complex as described in material and methods. The F_5_ peak showed an ability to destabilize the BlaI_2_-OP complex. F7 and F8 yielded also the same result. However, the K_av_ values calculated for F_7_ and F_8_ peaks were equal or higher than 1, meaning that only very small molecules could be present in these fractions. These fractions probably contain high concentration of salts that could be responsible for the destabilisation of the BlaI-operator complex during fluorescent EMSA (V. Duval, unpublished data).

**
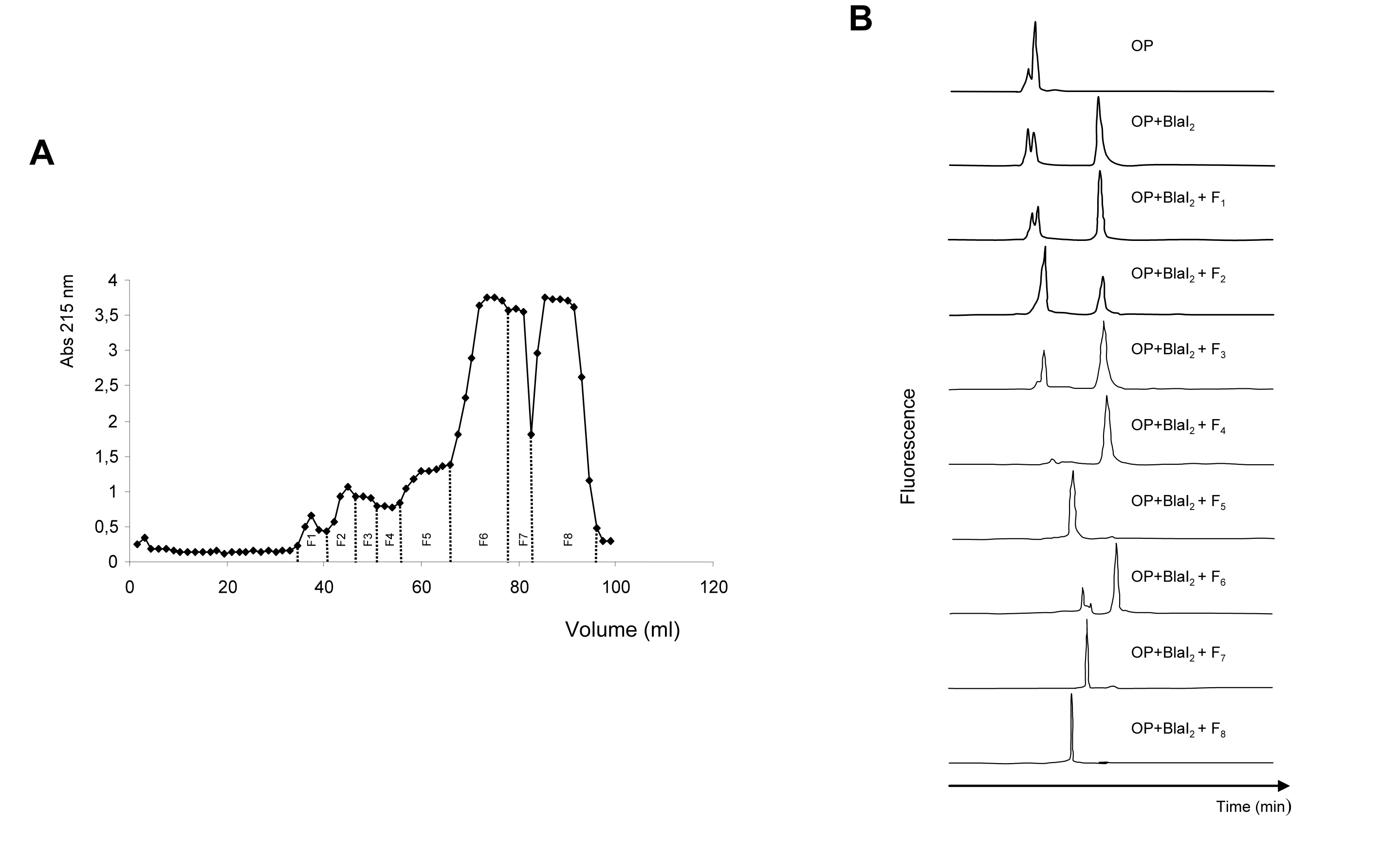
**


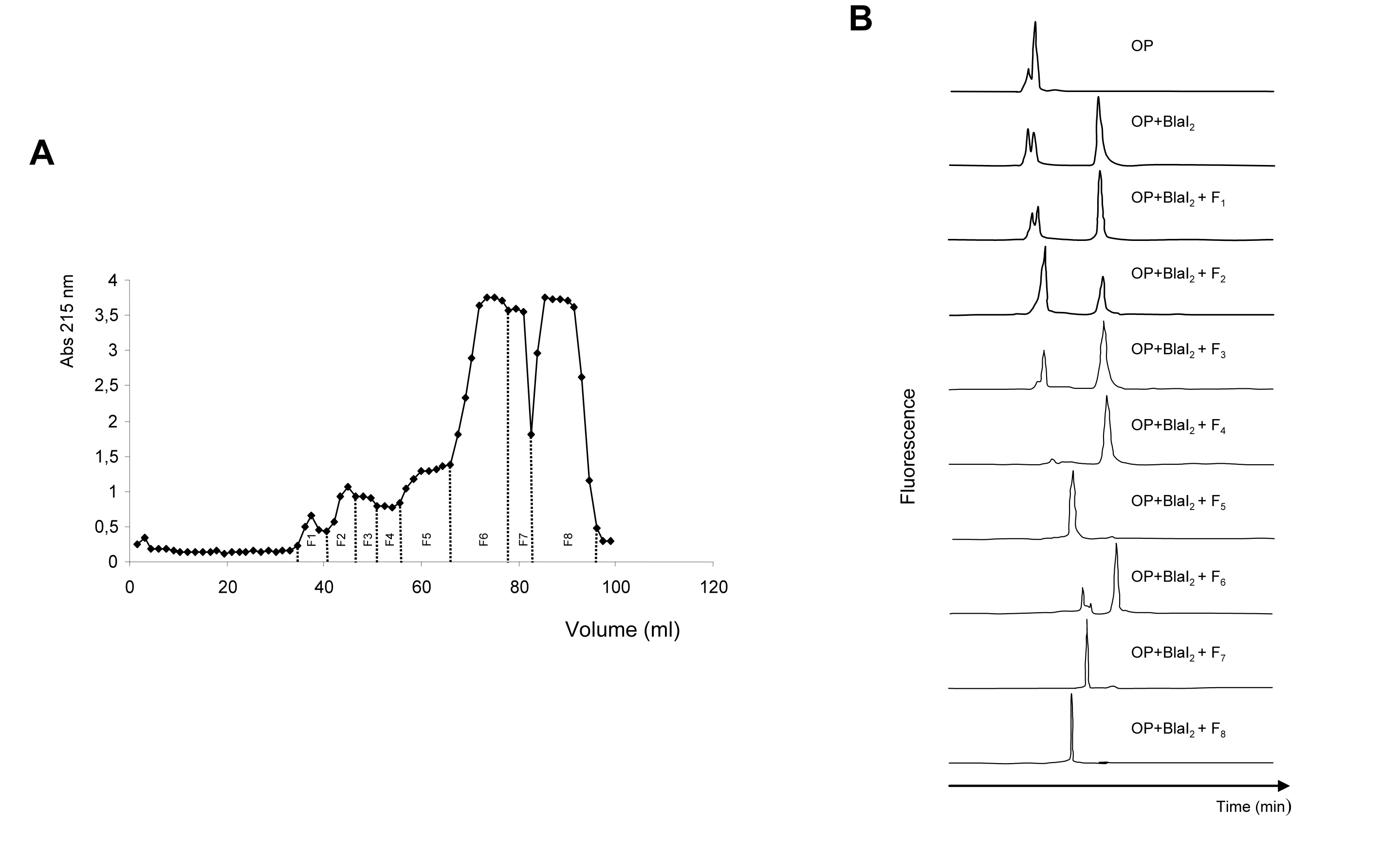


OP
%

BlaI_2_.OP

%

100

-

35

65

28

72

61

39

40

60

5

95

100

0

20

80

Eluted after the salt peak
and aberrant
electrophoretic mobility

**Figure S3: Identification of the coactivator in a cellular extract.**

(A) Enrichment of the active fraction obtained after molecular sieving fractionation.

The F_5_ peak from molecular sieving chromatography was treated to capture and to concentrate the coactivator: experiments involved a His-tagged BlaI (BlaI_His_)_2_ and Ni-NTA magnetic beads. First, the coactivator present in F_5_ was captured by incubating the (BlaI_His_)_2_.OP complex with F_5_. Then, the potentially present complexes, (BlaI_His_)_2_; (BlaI_His_)_2_.DNA and (BlaI_His_)_2_.coactivator were adsorbed onto Ni-NTA magnetic beads. Beads were pulled down with a magnet and the supernatant was collected (F_5_-1 fraction). The beads were incubated for 30 min at 55°C in phosphate buffer to release the coactivator and the supernatant was collected again (F_5_-2 fraction). The last step was repeated by resuspending beads in 5 mM phosphate buffer (pH 5.0) and the supernatant was collected as previously (F_5_-3 fraction).

(B) Fractions F_5_-1, F_5_-2, and F_5_-3 were then resuspended in 50 mM sodium borate (pH 9.5) for further 2,4,6-Trinitrobenzene Sulfonic Acid (TNBS) modification of peptides (Gevaert et al (2003)). Each TNBS modified fraction was freeze-dried, resuspended in 50 µl of 0.1% trifluoroacetic acid (TFA) and injected to a 100-5C-18ec (250 × 4.6 mm) column (Macherey-Nagel) for HPLC analysis.

The column was eluted at a flow rate of 0.7 ml**/**min with 0.1% TFA in water Milli-Q (2 min) followed by a linear gradient from 0 to 70% acetonitrile over 60 min**.** Chromatograms were obtained by following the absorbance at 335 nm. As expected, in the F_5_-2 fraction, a peak corresponding to the elution time of the dipeptide 1 (labelled by a cross) increased when compared with the F_5_-1 fraction. The same peak increased in the F_5_-3 fraction.

(C) To demonstrate that the peak of interest effectively corresponds to dipeptide 1, a small quantity of TNP-dipeptide 1 has been added to the TNBSA-modified F_5_-2 fraction. As expected, the TNP-dipeptide 1 co-eluted with the enriched peak in fraction F_5_-2 .

Reference

Gevaert, K. *et al.* Exploring proteomes and analyzing protein processing by mass spectrometric identification of sorted N-terminal peptides. *Nat Biotechnol* **21**, 566-569 (2003)

**A**

**Figure S4 BlaI/MecI repressors and the dipeptide using STD methods and chemical shift mapping by NMR**

(A) Full STD ^1^H spectrum performed on the of γ-D-Glu-*m-*A_2_pm after the addition of BlaI repressor at a [Ligand]/[Protein] ratio of 50. Due to the residual water signal, saturation transfer from the protein to the peptide is pointed out by the presence of resonances in the region between 0 and 4 ppm corresponding to the side chain proton of the dipeptide.

(B and C) ^15^N and ^1^H chemical shift variations observed on MecI in presence of two different dipeptides γ-L-Glu-L-Lys and γ-D-Glu-L-Lys.

The panel (B) shows the Sofast-HMQC experiments performed on MecI repressor with the control dipeptide addition. Spectrum of free MecI is plotted in red. Spectrum of MecI in presence of γ-L-Glu-L-Lys at a [Dipeptide]/[Protein] ratio of 50 is plotted in blue. The absence of chemical shift variation between the two spectra reveals that the control dipeptide does not interact significantly with the protein at this ratio.

The panel (C) shows a resolved region of the Sofast-HMQC experiments performed on MecI repressor upon ligand dipeptide addition. Spectrum of free MecI is plotted in red. Spectrum of MecI in presence of γ-D-Glu-L-Lys at a [Dipeptide]/[Protein] ratio of 50 is plotted in blue.

**Figure S5: Increased susceptibility to proteolysis of the MecI.dipeptide 2 complex showed by Mass spectra.**

(A) Mass spectra of MecI after a few hours of incubation at 25^o^C in 75 mM NaH_2_PO_4_/Na_2_HPO_4_ 300 mM KCl buffer at pH 7.6.

(B) Mass spectra of MecI after a few hours of incubation with the dipeptide 2 at 25^o^C in 75 mM NaH_2_PO_4_/Na_2_HPO_4_ 300 mM KCl buffer at pH 7.6.
([Dipeptide 2]/[MecI] ratio = 50)

In the case of MecI alone, the repressor integrity is maintained during few hours at 25^o^C (native MecI: 15,895 Da). On the contrary, dipeptide 2 addition mediates MecI destabilization that leads to increase repressor susceptibility to contaminant proteases present in the mixture (native MecI: 15,895 Da and fragments generated: 10,182 and 10,797 Da).

Mass spectra were acquired on a MALDI-TOF instrument (Autoflex, Bruker Daltonics). The samples (0.5 µl at 2.5 µM MecI) were mixed on the target with 0.5 µl sinapinic acid solution. Spectra were acquired in a linear mode over the 9000-30000 *m/z* range and processed using flexAnalysis (3.0) (Bruker Daltonics). An external mass calibration was applied using a mixture of insulin (5,733.5 Da), ubiquitin I (8,564.8 Da), cytochrome C (12,360.0 Da), myoglobin (19,651.3 Da) corresponding to the Protein Calibration Standard I from Bruker Daltonics.

**Figure S6: Peptidoglycan hydrolases found in Eubacteria.**

The peptidoglycan structure shown is that from *E. coli* or *B. subtilis.*

1: MurNAc-L-Ala-amidase; 2: γ-D-glutamyl-*m*-A_2_pm amidase; 3: L,D-carboxypeptidase; 4: D,D-carboxypeptidase; 5: *N*-acetyl-muramidase; 6: *N*-acetyl-glucosaminidase. To date, no hydrolase cleaving the L-Ala-D-Glu peptide bond (marked by ?) has been identified.

**Figure S7:** **Effect of the inactivation of *ykf*ABCD** **operon genes on the BlaP β-lactamase induction.**

The *Bacillus subtilis* mutants BFS1807 (*ykf*A^-^), BFS1808 (*ykf*B^-^), BFS1809 (*ykf*C^-^) and BFS1810 (*ykf*D^-^) present in the MICADO database were provided by Dr Kevin Devine from Trinity College, Dublin (Kobayashi et al (2003), Biaudet et al (1997)). The inactivation of each of the *ykf*ABCD genes was performed using a pMUTIN plasmid The integration of pMUTIN1 vector into the target gene has three consequences: (1) the targeted gene is inactivated; (2) *lacZ* becomes transcriptionally fused to the gene, allowing its expression pattern to be monitored; (3) the Pspac promoter controls the transcription of downstream genes in an IPTG-dependent fashion. The potential polar effects generated by the integration of the vectors can be alleviated by addition of 1 mM IPTG (Vagner et al (1998)). The presence of the insertions has been confirmed by PCR by using one primer complementary to the pSpac promoter and one complementary to the sequence downstream of the inactivated gene (see panel A).The wild-type strain was used as negative control. The four verified mutants were then transformed with plasmid pDML995 (Filée et al (2002)) to evaluate the effect of gene inactivation on BlaP β-lactamase induction. The transformants were respectively named BS995-ykfA^-^, BS995-ykfB^-^, BS995-ykfC^-^ and BS995-ykfD^-^. They were grown in LB medium supplemented with 7 µg/ml of chloramphenicol at 37°C until A^600^ reached 0.8. Then, cephalosporin C was added at a final concentration of 2.5 µg/ml. The same experiment was performed in the presence of 1 mM IPTG. After 0, 1, 2 and 3 hours of induction, samples were taken and A^600^ was measured. Beta-lactamase activity was determined by measuring nitrocefin hydrolysis (100 µM) at 482 nm. The BlaP quantity [E_t_] was calculated with following equations: v_0_ = (ΔA x s^-1^ x A^600-1^)/ε and v_0_ = (*k*_cat_ x [E_t_] x [S]) / (*K*_m_ + [S]) where v_0_ = first rate; ΔA = absorbance variation; *k*_cat_ = catalytic constant (470 s^-1^); [S] = substrate concentration (100 µM); *K*_m_ = 40 µM; ε = nitrocefin molar extinction coefficient (15000 M^-1^ cm^-1^).

(A) Integration of pMUTIN1 into the *ykfABCD* operon. pMUTIN1 (red box) was integrated in the target gene by a single crossing-over event. Broken arrows denote the promoter of Pspac induced by IPTG. Pspac promoter is strongly repressed by the *lacI* gene product carried on pMUTIN1. However, some residual expression can be accounted from this promoter. Arrows indicate PCR primers used in this study to verify the presence and the orientation of the different pMUTIN1 integrations.

(B, C, D and E) Induction of the BlaP β-lactamase by cephalosporin C (2.5 µ/ml) for the different *B. subtilis* mutants: BS995-ykfA^-^ (B), BS995-ykfB^-^ (C), BS995-ykfC^-^ (D) and BS995-ykfD^-^ (E) with or without IPTG (1 mM).

(○) non-induced BS995 (*B. subtilis* + pDML995, control strain); (●) induced BS995; (□) non-induced BS995 mutant; (■) induced BS995 mutant. In presence of IPTG, the genes under the control of Pspac promoter are fully expressed (*ykfA^-^BCD*, *ykfAB^-^CD*, *ykfABC^-^D*, *ykfABCD^-^*). On the contrary, without IPTG, the genes under the control of Pspac promoter are repressed (*ykfA^-^xBCD*, *ykfAB^-^xCD*, *ykfABC^-^xD*). However, some residual expression from this promoter can be accounted for some extent (Vagner et al (1998); Kobayashi et al (2003)). This promoter leakage could explain that no significant difference was observed with or without IPTG. For each strain, the data presented are the mean values obtained for three different clones.

Minimal inhibitory concentration (MIC) values for penicillin were also determined for each mutant strain carrying pDML995 and listed in the following table. The MIC values are in good agreement with those obtained for BlaP β-lactamase production: the *B. subtilis ykfA^-^* + pDML 995 is the more sensentive to penicillin and the lower β-lactamase producer.

| Strain | MIC to Penicillin  (µg/ml) |
| --- | --- |
| *B. subtilis* WT | 0.25 |
| *B. subtilis* WT+ pDML 995 | 5 |
| *B. subtilis ykfA^-^* + pDML 995 | 1.25 |
| *B. subtilis ykfB^-^* + pDML 995 | 2.5 |
| *B. subtilis ykfC^-^* + pDML 995 | 2.5 |
| *B. subtilis ykfD^-^* + pDML 995 | 2.5 |

References

Biaudet, V., Samson, F. & Bessieres, P. Micado--a network-oriented database for microbial genomes. *Comput Appl Biosci* **13**, 431-438 (1997).

Kobayashi, K. *et al.* Essential *Bacillus subtilis* genes. *Proc Natl Acad Sci U S A* **100**, 4678-4683 (2003).

Vagner, V., Dervyn, E. & Ehrlich, S. D. A vector for systematic gene inactivation in *Bacillus subtilis*. *Microbiology* **144**, 3097-3104 (1998).

Filée, P. *et al.* The fate of the BlaI repressor during the induction of the *Bacillus licheniformis* BlaP beta-lactamase. *Mol Microbiol* **44**, 685-694 (2002).

Figure S7

A


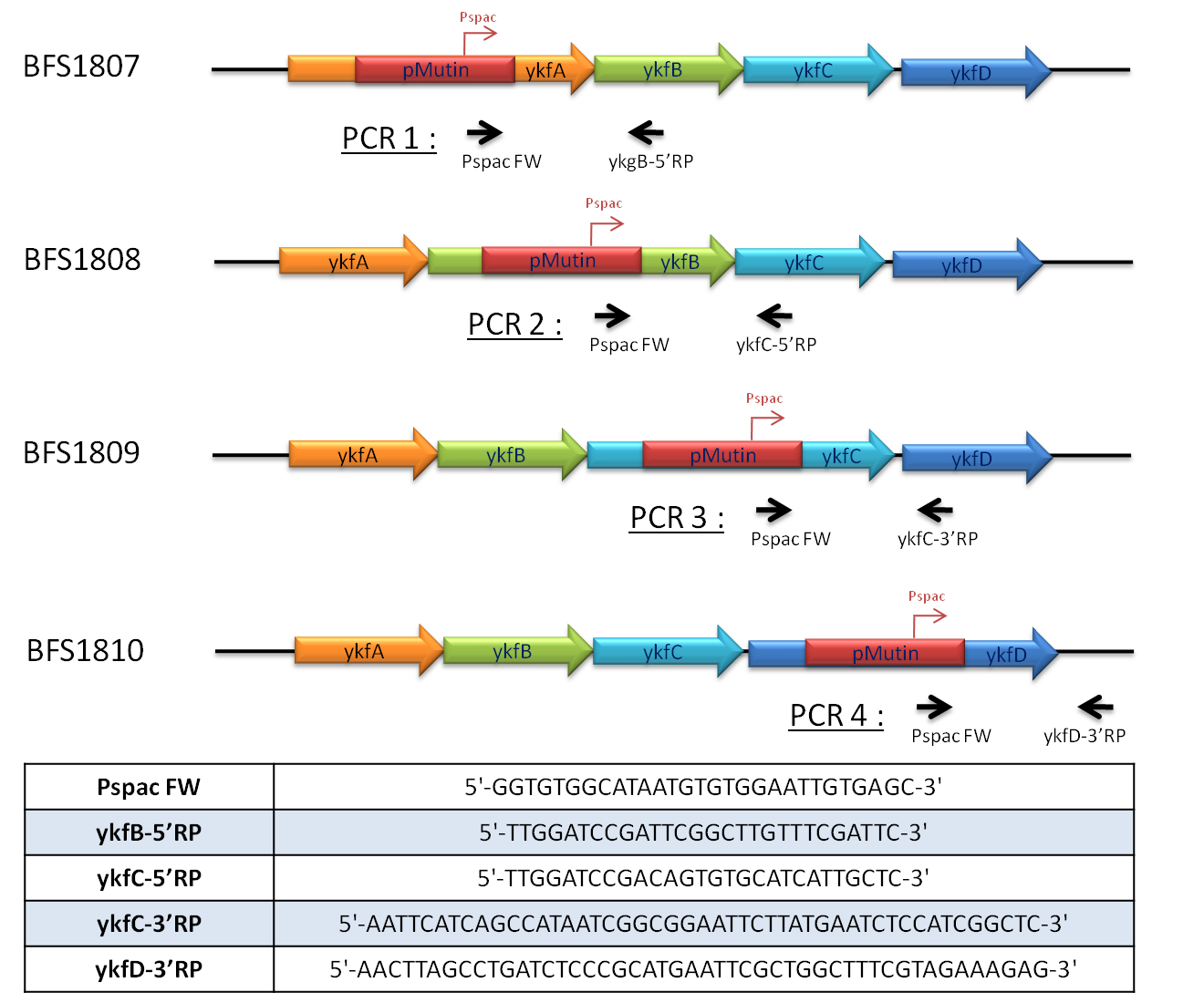


Figure S7 B, C, D and E

Organic synthesis of dipeptide coactivators

γ-D-Glu-*m*A_2_pm

NMR 1H (D_2_O, 400 MHz) and ESI MS have been recorded.

NMR (400MHz, D_2_O) d 4.33 (1H, m), 3.99 (2H, m), 2.50 (2H, m), 2.17 (2H, m), 1.76-1.90 (4H, m) 1.47 (2H, m)

Mass spectrum was recorded with a Finnigan TSQ7000 mass spectrometer (ThermoElectronCorp.) operating in full-scan MS mode with an ESI+ source: 320 (M+1)

The NMR spectrum is similar to the one described by A. Chowdhury and G.-J. Boons in their [Tetrahedron Letters](http://www.sciencedirect.com/science/journal/00404039), 46,1675-1678 (2005) paper.

^1^H NMR (500 MHz, CD_3_OD): *δ* 4.32 (1H, q, α-CH, DAP), 3.94 (1H, t, α-C*H*, Glu), 3.86 (1H, q, α-C*H*, DAP), 2.47 (2H, γ-C*H*_2_, Glu), 2.10–2.19 (1H, m, β-C*H*_2_, Glu), 2.04–2.10 (1H, m, β-C*H*_2_, Glu), 1.78–1.96, 1.65–1.69, 1.45–1.57 (6H, m, β,γ,δ-C*H*_2_C*H*_2_C*H*_2_, DAP)

γ-D-Glu-L-Lys

NMR 1H (D_2_O, 500 MHz) and ESI MS have been recorded.

^1^H NMR (500 MHz, D_2_O): *δ* 4.29 (1H, q, α-CH, Lys), 3.94 (1H, t, α-C*H*, Glu), 2.92 (2H, t, H2N-C*H_2_*, Lys), 2.45 (2H, γ-C*H*_2_, Glu), 2.10–2.19 (2H, m, β-C*H*_2_, Glu), 1.80-1.88, 1.68-1.74 (2H, m, β-C*H*_2_, Lys), 1.58–1.66, (2H, m, δ-CH_2_, Lys), 1.36–1.42 (2H, m, γ-C*H*_2_, Lys).

^13^C NMR (100MHz, D_2_O): 178.4, 177, 174.5, 55.3, 51.6, 41.9, 33.4, 32.6, 28.9, 28.2 24.7

Mass spectrum was recorded with a Finnigan TSQ7000 mass spectrometer (ThermoElectronCorp.) operating in full-scan MS mode with an ESI+ source: 276 (M+1)

Furthermore, three different sources of dipeptide were utilised for this study: the natural one, obtained from peptidoglycan digestion (γ-DGlu-*m*A_2_p), chemically synthesised by N. Teller γ-DGlu-*m*A_2_p and γ-D-Glu-L-Lys, and finally, a customer synthesised γ-D-Glu-L-Lys (Genecust, Luxembourg). In all the cases, the result was the same.
